# Effect of prohibiting the use of Paraquat on pesticide-associated mortality

**DOI:** 10.1186/s12889-017-4832-4

**Published:** 2017-11-02

**Authors:** Jinyong Kim, Sang Do Shin, Seungmin Jeong, Gil Joon Suh, Young Ho Kwak

**Affiliations:** 1 Seoul National University Hospital, Seoul, South Korea; 2 Seoul National University College of Medicine, Seoul, South Korea; 3 Seoul National University Graduate School of Public Health, Seoul, South Korea

**Keywords:** Paraquat, Mortality, Suicide, Public intervention

## Abstract

**Background:**

Paraquat is associated with a high rate of fatalities in acute poisoning. This study aimed to examine the association between the national public health policy that banned the use of paraquat and the incidence of pesticide-associated mortality.

**Methods:**

All external causes of death from 2009 to 2013 of Korea were analyzed. The intervention was a national public health policy that annulled the authorized use (2011) and banned the purchase of paraquat (2012). Two periods were compared as follows: before (2009-2010) and after (2012-2013) the intervention period. The main outcome was pesticide-associated death coded on the death certificate. Multivariable logistic regression analysis adjustment for gender, age, season and weekday of death, province, education level, marital status, and occupation was performed to calculate adjusted odds ratios (AORs) and 95% confidence intervals (CIs) for pesticide-associated mortality. The effect sizes of the intervention across all intents (Accident, Suicide, Homicide, and Undetermined) were compared by adding an interaction term (intervention*intent group) to the above model.

**Results:**

A total of 127,866 deaths from for all external causes were analyzed, including 65,538 from 2009 to 2010 and 62,373 from 2012 to 2013. Pesticide-associated mortality decreased from 9.7% (2009-2010) to 6.5% (2012-2013) (*p* < 0.001). The AOR (95% CI) of the intervention on pesticide-associated mortality was 0.59 (0.56-0.62). The AORs of the intervention according to intent were 0.72 (0.55-0.96) in the Accident group, 0.61 (0.58-0.64) in the Suicide group, 1.29 (0.43-3.87) in the Homicide group, and 0.44 (0.38-0.50) in the Undetermined group.

**Conclusion:**

The national public health policy that banned paraquat resulted in a significant decrease in pesticide-associated mortality.

## Background

According to WHO reports in 2014, suicide is a critical public health issue that leads to over 800,000 deaths every year. One of the most frequently used means of suicide is self-poisoning by deliberate ingestion of effective pesticides or herbicides that are lethal to human beings [1–4]. According to the World Health Organization (WHO) mortality database, pesticide poisoning is common in many Asian countries, while drug poisoning is more rampant in Northern Europe and the United Kingdom [5]. According to Statistics Korea, more than half of poisoning deaths are due to pesticide or herbicide ingestion [3]. Among agrochemical products, paraquat was the most widely used for farming and was considered to be the main lethal suicide agent in South Korea [2, 6, 7]. Although it is relatively safe when used appropriately, paraquat ingestion leads to development of intracellular oxidative stress in the lungs and other organs, resulting in death within a few days [8]. No known medical treatments, including activated charcoal, gastric lavage, hemodialysis, and drugs such as immunosuppressive agents and antioxidants, have been proven to reduce mortality in emergency care setting [9]. Case fatalities from acute paraquat poisoning exceed 70% [1], and according to Statistics Korea, paraquat resulted in 3206 deaths in 2010.

It is well known that restricting easy access to means of self-harm is effective in reducing mortality from suicide [5, 10, 11]. In an effort to reduce accidental and incidental poisoning, the Government of the Republic of Korea has consistently implemented national interventions. In 1999, the Act on Paraquat Regulations labeled paraquat products as “Restricted use products (RUPs)”, with policy revisions made in 2005. However, although accidental ingestion decreased, intentional self-poisoning actually increased to over 2000 deaths in 2009. As a final countermeasure, the government annulled the direct authorization of all paraquat products in November 2011 and banned the use of paraquat in October 2012 [12].

Policies banning or restricting the use of paraquat have been enforced in many countries, but there are few data evaluating their effectiveness in decreasing the numbers of deaths from self-poisoning. Moreover, a study from Marseille, France showed that the number of suicide attempts involving paraquat was unchanged despite the ban imposed by the European Union in July 2007 [13]. In contrast, when Western Samoa experienced fluctuations in paraquat importation from 1972 to 1987 due to national financial problems, paraquat use and self-poisoning showed a similar pattern of fluctuations [14, 15]. This phenomenon suggests that paraquat availability, in addition to the existence of nationwide campaigns raising suicide awareness, is an important factor that influences suicide-related deaths. Paraquat poisoning, whether accidental or intentional, is fatal. The public health policy banning both the sale and the production of paraquat aimed to reduce the number of accidental and intentional poisonings. However, it is unclear whether the policy has been successful. The heterogeneity of agricultural systems, social structures, and governmental restrictions among different countries invariably leads to varying outcomes from national interventions.

This study aimed to evaluate the effect of the national banning of paraquat on the number of PESTICIDE-associated deaths and to compare the size of the effect based on the intent of the poisoning.

## Methods

The study was exempted by the Institutional Review Board because the database contained no private information and was open to the public.

### Study design and setting

This was a before- and after-intervention observational trial in Korea, a country with a population of approximately 50 million people.

### Areas of paraquat use

According to the Korea Crop Protection Association guideline, paraquat should have been used only in orchards, mulberry trees, and forest areas. The Korea Crop Protection Association guideline strictly prohibited its application around habitats, schools, recreational areas, playgrounds, waysides, and golf courses. However, the bulk of paraquat use occurred on farmlands and in urban settings, with inappropriate applications. For example, paraquat was commonly applied days before sowing rice and vegetables and during winter on golf courses.

Agrochemical registration and management scheme in the Republic of Korea.

In 1957, the Agrochemicals Control Act (ACA) was initially promulgated as a permission and notification system. The ACA allowed massive registration of pesticide commodities with low technical grades of active substances (TGAIs), including paraquat. In 1996, the ACA was amended to function as a full registration scheme with comprehensive reviews on pesticide efficacy, phytotoxicity, toxicity, residue tests and reregistration every ten-years [16]. Paraquat was imported in 1970 without any restrictions on its use. In 1999, the Act on Paraquat Regulations labeled paraquat products as “Restricted use products (RUPs)” and awarded licenses only to farmers who completed “agrochemical safe use education”. This education, however, could be offered by the Municipal Agriculture Technology Center, agrochemical sellers, or even village leaders. Due to incomplete, lenient, and indefinite enforcement of safety education, the license remained nominal [12].

### Agrochemical use and market

Chemical control is currently the most widely used technique among weed control methods. Due to labor shortages in an already shrinking farming population, weed removal mainly depends on the massive spraying of cheap herbicides. Agrochemical use has recently decreased as follows: 13.0 kg/ha, 1.9 kt in 2004 to 10.9 kg/ha, 1.7 kt in 2013. According to the Agrochemical Yearbook, in 2012, the agrochemical market size was approximately 1.2 trillion won (1.08 billion dollars), of which nonselective herbicide represented 8.3%. Paraquat products represented 39% of nonselective herbicides, which is approximately 40.3 billion won (36 million dollars), ranking among the top 1467 registered agrochemicals.

### Data source

According to the Act on Family Relation Registration, all deaths must be reported within one month through the Notice of Death. After death is confirmed, a death certificate is completed by a physician and includes information regarding age, gender, education level, marital status, occupation, administrative district of residence, place of death, cause of death, intentionality of death, and date of death. The cause of death is recorded on three levels as follows: the direct, proceeding, and underlying cause. Following completion of the death certificate, close relatives or a certified facility or program where the death occurred submit the death certificate and death notification to local registrars. In the process of data registration, the external cause of injury, when present, is coded based on the International Classification of Diseases and Related Health Problems, 10th Revision (ICD-10). Pesticide poisoning deaths are defined as those featuring the code for toxic effects of pesticides (T600–T609). Death data are registered in the National Vital Statistics System, which is eventually collected by the National Statistics Office of Korea and released to the public annually. Foreigners are not included, although persons with dual nationality are included. In 2010, 590,000 people, or 1.2% of the total population, were foreigners.

### Selection of study participants

All cases from the National Death Certificates were classified as accidents, suicides, homicides, legal interventions, and undetermined causes. All deaths due to external causes between 2009 and 2013 were extracted from the database. There were no cases with missing information. We excluded patients who died in 2011, when the public health policy was implemented.

### Main intervention and exposure

The Rural Development Association sent a notification (2011-45) that annulled the direct authorization of all paraquat products in November 2011 and banned the use of paraquat in October 2012. A total of 11 paraquat dichloride liquid products were banned. The before-intervention period was from 2009 to 2010. The implementation period was in 2011, and the after-intervention period, which was used for comparison, was from 2012 to 2013. There was no public policy implementation during the study periods on banning the other pesticides.

### Additional covariates

Additional variables obtained from the National Death Certificates included age, gender, education level, marital status, occupation, administrative district of residence, place of death, cause of death, intentionality of death, and date and time of death. Age groups were defined as 0-9, 10-19, 20-29, 30-39, 40-49, 50-59, 60-69, and 70 years old and above. Residence was defined as metropolitan or non-metropolitan. Metropolitan was defined as having a population of more than one million. Occupation was classified as white collar, blue collar, or unemployed. White collar included managers, professionals and associates, and clerical support workers. Blue collar included service and sales workers; skilled agricultural, forestry and fishery workers; technicians and associate professionals; engineers; plant and machine operators and assemblers; and elementary occupations. The other/unknown group included students, housewives, unemployed persons, and unknown occupations. Marital status was defined as unmarried, married, divorced, deceased (widow/widower), and unknown. Education level was defined as elementary (6 years) and below, junior high school (3 years), senior high school (3 years), college and above high school, or unknown. Intent of death was classified as accident, suicide, homicide, and undetermined.

### Outcome measurements

The primary outcome was the total number of deaths due to pesticides poisoning, which was measured according to the ICD-10 classification (T60, Toxic effect of pesticides). Organophosphate and carbamate insecticides, halogenated insecticides, chlorinated hydrocarbons, other insecticides, herbicides and fungicides, rodenticides, other pesticides, and unspecified pesticide were classified into the same category due to difficulty in identifying the exact agent in clinical and administrative settings. Deaths due to specific agents were indistinguishable from and were treated as deaths due to pesticides.

### Statistical analysis

Demographic findings between the before- and after-intervention periods were compared. The characteristics among the intent groups (Accident, Suicide, Homicide, and Undetermined) were also compared.

The adjusted odds ratio (AOR) and 95% confidence interval (95% CI), with respect to the intervention (after- versus before-intervention) and pesticide-associated mortality, were calculated using multivariable logistic regression to estimate the effect of the intervention. Adjusted risk factors applied in the multivariable logistic regression model included age, gender, residence, death season, death weekday, occupation, marital status, education level, and intent of death.

To compare the effect size of the intervention according to intent group, an interaction model was used by adding the interaction term (intervention*intent group) to the final multivariable logistic regression model.

## Results

### Demographic findings

**Fig. 1:**
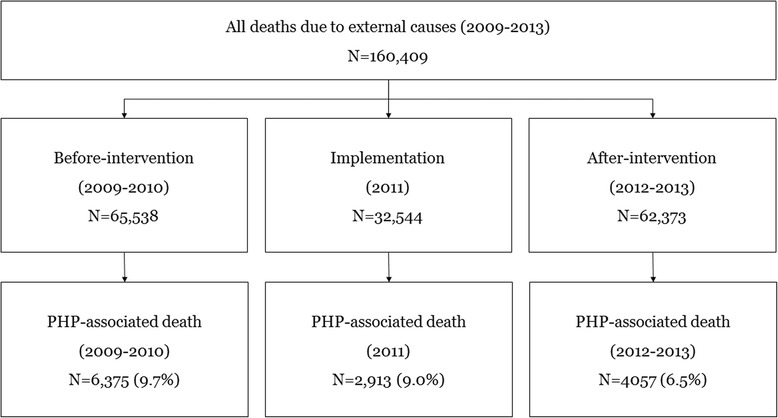
Study population. PP: pesticide poisoning

**Fig. 2:**
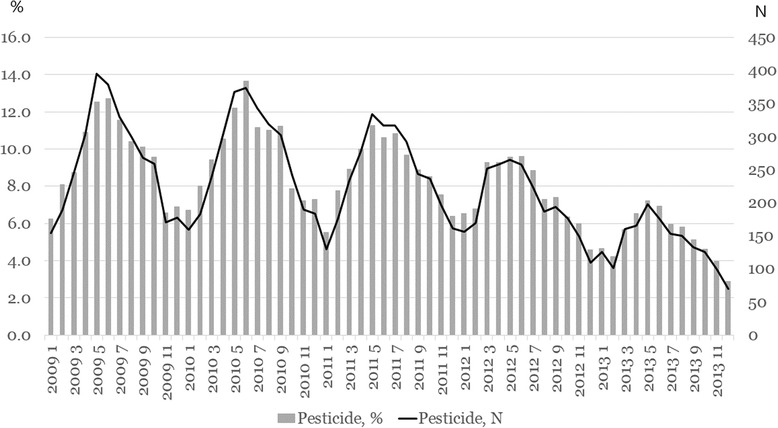
Proportions and number of pesticide-associated mortality among all deaths due to external causes by year and month

A total 127,866 deaths from all external causes were analyzed during the study periods, including 65,538 from 2009 to 2010 and 62,373 from 2012 to 2013 (Fig. 1). The proportions of mortality by pesticide- and herbicide-associated mortality among total mortality by year and month has been decreased after 2011 (Fig. 2). The distribution of risk factors (gender, age group, metropolis, season of death, occupation, marital status, education level, and intent) was significantly different between the two periods (Table 1). pesticide-associated was responsible for 9.7% of deaths in the before-intervention period and 6.5% of deaths in the after-intervention period (*p* < 0.0001). Among the intent groups, the distribution of risk factors was significantly different. The pesticide-associated mortality rate was 0.4% in the Accident group, 15.1% in the Suicide group, 0.5% in the Homicide group, and 10.5% in the Undetermined group (p < 0.0001) (Table 2).

**Table 1:** Comparison of demographic characteristics of the study population between the before- and after-intervention periods

Factors	All		Before-intervention 2009-2010		After-intervention 2012-2013		*P*-value
	N	%	N	%	N	%	
All	127,866	100.0	65,504	100.0	62,362	100.0	
Gender							0.0378
Female	41,333	32.3	21,348	32.6	19,985	32.0	
Male	86,533	67.7	44,156	67.4	42,377	68.0	
Age, years							<0.001
0-9	1294	1.0	714	1.1	580	0.9	
10-19	3183	2.5	1819	2.8	1364	2.2	
20-29	9621	7.5	5627	8.6	3994	6.4	
30-39	14,349	11.2	7807	11.9	6542	10.5	
40-49	20,375	15.9	10,909	16.7	9466	15.2	
50-59	22,188	17.4	10,832	16.5	11,356	18.2	
60-69	18,249	14.3	9727	14.8	8522	13.7	
70-	38,607	30.2	18,069	27.6	20,538	32.9	
Residence							0.0217
Metropolis	48,578	38.0	25,085	38.3	23,493	37.7	
Non-metropolis	79,288	62.0	40,419	61.7	38,869	62.3	
Season							<0.001
MAR-MAY	33,502	26.2	17,160	26.2	16,342	26.2	
JUN-AUG	32,923	25.7	17,424	26.6	15,499	24.9	
SEP-NOV	32,073	25.1	16,356	25.0	15,717	25.2	
DEC-FEB	29,368	23.0	14,564	22.2	14,804	23.7	
Weekday							0.4011
Sunday	17,478	13.7	9027	13.8	8451	13.6	
Monday	19,268	15.1	9919	15.1	9349	15.0	
Tuesday	18,560	14.5	9410	14.4	9150	14.7	
Wednesday	18,437	14.4	9354	14.3	9083	14.6	
Thursday	18,092	14.1	9310	14.2	8782	14.1	
Friday	18,136	14.2	9285	14.2	8851	14.2	
Saturday	17,895	14.0	9199	14.0	8696	13.9	
Occupation							<0.001
White collar	14,611	11.4	7693	11.7	6918	11.1	
Blue collar	39,449	30.9	19,580	29.9	19,869	31.9	
Unemployed^a^	73,806	57.7	38,231	58.4	35,575	57.0	
Marital status							<0.001
Unmarried	28,516	22.3	15,062	23.0	13,454	21.6	
Married	61,820	48.3	31,963	48.8	29,857	47.9	
Divorced	16,454	12.9	9168	14.0	7286	11.7	
Deceased	20,753	16.2	9152	14.0	11,601	18.6	
Unknown	323	0.3	159	0.2	164	0.3	
Education level							<0.001
Elementary school and below	48,501	37.9	25,230	38.5	23,271	37.3	
Middle school	19,284	15.1	9905	15.1	9379	15.0	
High school	38,445	30.1	19,740	30.1	18,705	30.0	
College and above	20,215	15.8	10,145	15.5	10,070	16.1	
Unknown	1421	1.1	484	0.7	937	1.5	
Intent							<0.001
Accident	54,313	42.5	27,073	41.3	27,240	43.7	
Suicide	59,713	46.7	31,051	47.4	28,662	46.0	
Homicide	2427	1.9	1315	2.0	1112	1.8	
Undetermined	11,413	8.9	6065	9.3	5348	8.6	
Pesticide							<0.001
Not-associated	117,434	91.8	59,129	90.3	58,305	93.5	
Associated	10,432	8.2	6375	9.7	4057	6.5	

^a^Unemployed group includes students, housewives, military personnel, and unemployed persons

**Table 2:** Comparison of demographic characteristics among the intent of injury groups

Factors	All		Accident		Suicide		Homicide		Unknown		p-value
	N	%	N	%	N	%	N	%	N	%	
All	127,866	100.0	54,313	100.0	59,713	100.0	2427	100.0	11,413	100.0	
Gender											<.0001
Female	41,333	32.3	16,769	30.9	19,681	33.0	1111	45.8	3772	33.1	
Male	86,533	67.7	37,544	69.1	40,032	67.0	1316	54.2	7641	66.9	
Age, years											<.0001
0-9	1294	1.0	987	1.8	5	0.0	235	9.7	67	0.6	
10-19	3183	2.5	1488	2.7	1445	2.4	120	4.9	130	1.1	
20-29	9621	7.5	3022	5.6	5986	10.0	200	8.2	413	3.6	
30-39	14,349	11.2	3781	7.0	9622	16.1	292	12.0	654	5.7	
40-49	20,375	15.9	7086	13.0	11,423	19.1	612	25.2	1254	11.0	
50-59	22,188	17.4	9134	16.8	10,961	18.4	531	21.9	1562	13.7	
60-69	18,249	14.3	8401	15.5	7809	13.1	213	8.8	1826	16.0	
70-	38,607	30.2	20,414	37.6	12,462	20.9	224	9.2	5507	48.3	
Residence											<.0001
Metropolis	48,578	38.0	18,272	33.6	25,128	42.1	1023	42.2	4155	36.4	
Non-metropolis	79,288	62.0	36,041	66.4	34,585	57.9	1404	57.8	7258	63.6	
Season											<.0001
MAR-MAY	33,502	26.2	13,061	24.0	16,780	28.1	609	25.1	3052	26.7	
JUN-AUG	32,923	25.7	13,497	24.9	15,996	26.8	690	28.4	2740	24.0	
SEP-NOV	32,073	25.1	14,396	26.5	14,279	23.9	566	23.3	2832	24.8	
DEC-FEB	29,368	23.0	13,359	24.6	12,658	21.2	562	23.2	2789	24.4	
Weekday											<.0001
Sunday	17,478	13.7	7633	14.1	7865	13.2	340	14.0	1640	14.4	
Monday	19,268	15.1	7811	14.4	9396	15.7	421	17.3	1640	14.4	
Tuesday	18,560	14.5	7704	14.2	8852	14.8	359	14.8	1645	14.4	
Wednesday	18,437	14.4	7689	14.2	8755	14.7	345	14.2	1648	14.4	
Thursday	18,092	14.1	7645	14.1	8538	14.3	305	12.6	1604	14.1	
Friday	18,136	14.2	7757	14.3	8450	14.2	331	13.6	1598	14.0	
Saturday	17,895	14.0	8074	14.9	7857	13.2	326	13.4	1638	14.4	
Occupation											<.0001
White collar	14,611	11.4	6278	11.6	7306	12.2	306	12.6	721	6.3	
Blue collar	39,449	30.9	20,942	38.6	14,846	24.9	749	30.9	2912	25.5	
Unemployed^a^	73,806	57.7	27,093	49.9	37,561	62.9	1372	56.5	7780	68.2	
Marital status											<.0001
Unmarried	28,516	22.3	10,029	18.5	15,969	26.7	842	34.7	1676	14.7	
Married	61,820	48.3	27,237	50.1	28,118	47.1	930	38.3	5535	48.5	
Divorced	16,454	12.9	6419	11.8	8061	13.5	375	15.5	1599	14.0	
Deceased	20,753	16.2	10,538	19.4	7414	12.4	267	11.0	2534	22.2	
Unknown	323	0.3	90	0.2	151	0.3	13	0.5	69	0.6	
Education level											<.0001
Elementary school and below	48,501	37.9	24,889	45.8	17,054	28.6	816	33.6	5742	50.3	
Middle school	19,284	15.1	8047	14.8	9197	15.4	353	14.5	1687	14.8	
High school	38,445	30.1	13,899	25.6	21,134	35.4	889	36.6	2523	22.1	
College and above	20,215	15.8	6930	12.8	11,691	19.6	339	14.0	1255	11.0	
Unknown	1421	1.1	548	1.0	637	1.1	30	1.2	206	1.8	
Intervention											<.0001
Before	65,504	51.2	27,073	49.8	31,051	52.0	1315	54.2	6065	53.1	
After	62,362	48.8	27,240	50.2	28,662	48.0	1112	45.8	5348	46.9	
Pesticide											<.0001
Not-associated	117,434	91.8	54,108	99.6	50,703	84.9	2414	99.5	10,209	89.5	
Associated	10,432	8.2	205	0.4	9010	15.1	13	0.5	1204	10.5	

^a^Unemployed group includes students, housewives, military personnel, and unemployed persons

### Main findings and interaction analysis

The AOR (95% CI) for pesticide-associated mortality with respect to the intervention was 0.59 (0.56-0.62) in the final model (Table 3). The AORs (95% CIs) were 98.35 (85.39-113.29) in the Suicide group, 2.96 (1.68-5.22) in the Homicide group, and 33.40 (28.71-38.87) in the Undetermined group compared with the Accident group.

**Table 3:** Multivariable logistic regression on pesticide-associated mortality by intervention and intent

Exposure	Total	Pesticide-associated death		Unadjusted			Adjusted^a^		
	N	N	%	OR	95% CI		OR	95% CI	
Total	127,866	10,432	8.2						
Intervention									
Before (2009-2010)	65,504	6375	9.7	1.00			1.00		
After (2012-2013)	62,362	4057	6.5	0.65	0.62	0.67	0.59	0.56	0.62
Intent									
Accident	54,313	205	0.4	1.00			1.00		
Suicide	59,713	9010	15.1	46.90	40.82	53.90	98.35	85.39	113.29
Homicide	2427	13	0.5	1.42	0.81	2.49	2.96	1.68	5.22
Undetermined	11,413	1204	10.5	31.13	26.80	36.15	33.40	28.71	38.87

*OR* odds ratio, *95% CI* 95% confidence interval

^a^adjusted for gender, age, season, weekday, residence, job, marital status, education level, intervention, and intent

The AORs (95% CIs) with respect to the intervention for the Accident, Suicide, Homicide, and Undermined groups in the interaction analysis were 0.72 (0.55-0.96), 0.61 (0.68-0.64), 1.29 (0.43-3.87), and 0.44 (0.38-0.50), respectively (Table 4).

**Table 4:** Effect of the intervention according to intent group on pesticide-associated mortality using multivariable logistic regression analysis in an interaction model

Intervention effect according to	Adjusted^a^		
	OR	95% CI	
Intent			
Accident	0.72	0.55	0.96
Suicide	0.61	0.58	0.64
Homicide	1.29	0.43	3.87
Undetermined	0.44	0.38	0.50

*OR* odds ratio, *95% CI* 95% confidence interval

^a^adjusted for gender, age, season, weekday, residence, job, marital status, education level, intent, and interaction term (intervention*intent)

## Discussion

This study showed the national public health intervention reduced mortality due to pesticide poisoning. In particular, both accidental, suicide, and undetermined death due to pesticide poisoning decreased, although there was not effective in the change in homicide poisoning. This public health intervention targeted the use of paraquat in an effort to reduce pesticide-associated accidental deaths and suicides. Interaction analysis revealed that the intervention also had a favorable effect on the pesticide-associated death rate in the undetermined group.

In South Korea, the suicide rate has been steadily increasing and was reported to be 31.7 per 100,000 persons in 2011 by the National Statistics Office of Korea, far exceeding the annual global age-standardized suicide rate of 11.4 per 100,000 persons. Suicides in Korea reached their peak in 2011. To impede the rising rate of suicide, the Korean government annulled the authorization of paraquat products and their use in 2011. A paper analyzed the suicide death during 2003 to 2013 for testing the association between public health regulation on paraquat and suicide mortality in Korea. The study included the suicide patients as study subject, excluding non-suicidal pesticide deaths [17]. We included all kinds of pesticide death regardless of suicide intent during study period. About 14% of pesticide death was associated with non-suicide death such as accidental death, homicide, and undetermined death for intent in our study. We tested the association between the intervention and pesticide mortality using a multivariable model. In addition, we tested the interactive effect between the intervention and injury intent on outcomes. We found the homicide was not associated with homicide, however most pesticides poisoning death were influenced by the public policy.

Cha ES, et al. showed a change in the secular trend for the suicide death due to pesticide. The pesticide-associated suicide has been decreasing and the banning policy reduce much more use of pesticide for suicide death [17]. Figure 2 showed the trend in proportions and total number of pesticide death among all kinds of injury death during 2009-2010 versus 2012-2013. Before intervention, the trend was not significant but after 2011, the decrease was much greater in both proportion and total number of pesticide-associated death. However, we did not calculate the age-gender standardized mortality due to pesticide during the period.

Although results from previous studies have been controversial regarding the effectiveness of the national intervention ban or reductions in the toxicity of pesticides, there is universal agreement that open access to pesticides is one of the most important factors contributing to poison-related deaths [5]. The agricultural region of Sri Lanka is characterized by paddy cultivation and high pesticide use. A survey of 669 patients from a district hospital found that 46% used pesticides due to their easy accessibility and that 43% chose popular suicide agents in their village [10]. In a previous study of 268 self-poisoning patients in rural Sri Lanka, 85% also cited easy availability as a determining factor in the chosen means of suicide, and more importantly, more than 50% impulsively harmed themselves with little knowledge regarding the lethality of the chosen substance or the treatment options for its effects [11]. Although Syngenta produced a new paraquat formulation named “Gramoxone INTEON” in an effort to mitigate the rising suicide rate, the company failed to demonstrate any benefits of the new product with respect to survival rates following pesticide-associated suicide attempts in Sri Lanka. It has been suggested that a large group of patients with low paraquat concentrations and a high survival rate (86.7%) was excluded from the study because the formulation was unidentifiable [18]. The withdrawal of paraquat by the European Union in 2007 had contrasting results in different areas. Pesticide-associated mortality in France, which was mostly unintentional, decreased after the ban although the sample size was too small to conclude the effect. The study data showed a marginal decline in total number of poisonings observed after the paraquat ban (38 vs 33 after the ban) mostly due to a decrease in the number of unintentional exposure (21 vs 16 after the ban). In contrast, in foreign French territories, such as French Guiana, which are highly depended on agriculture and illegally purchase paraquat from neighboring countries, the withdrawal policy was ineffective in decreasing access to lethal pesticides and, consequently, the number of pesticide-associated suicide attempts [13]. Japan was successful in controlling overall pesticide-associated mortality through their national intervention, although the level of poisoning in rural areas remained highly lethal. Pesticide-associated deaths decreased after the 1985 Advisory Resolution on Paraquat Regulations by the Japanese Association of Rural Medicine reduced the concentration of paraquat products from 24% to 5% and subsequently suspended paraquat production [19]. Although overall pesticide-associated mortality decreased with the modified paraquat product, its effect on rural poisoning remained subtle. In a study of hospitals affiliated with the Japanese Association of Rural Medicine from 1998 to 2002, paraquat was responsible for 20% of pesticide-associated deaths, and suicide was responsible for 70% of pesticide-associated deaths from a total of 346 pesticide poisoning cases. The death rate from poisoning still accounted for more than 70% of clinical cases [20]. Recent data from the Japanese Association of Rural Medicine showed that the death rate from suicide attempts with paraquat remained at approximately 80%, indicating that the low-concentration paraquat product was not an effective alternative for reducing pesticide-associated mortality in rural areas [21].

Some national interventions had no impact on reducing pesticide-associated mortality by banning inadequate pesticides/herbicides. In Taiwan, the age-standardized rate of pesticide-related suicide declined 67%, from 7.7 per 100,000 persons (42% of all suicides) in 1987 to 2.5 per 100,000 persons (12% of all suicides) in 2010, despite a 69% increase in suicide rates by other methods. Pesticide ingestion was the most commonly used means of suicide in 1987; however, hanging and non-domestic gas poisoning became more prevalent by 2010. The decrease was coincident with a 66% decline in the agricultural working population, although the correlation between the bans on selected pesticide products and the decrease in pesticide-associated suicides was weak. The bans were primarily implemented *after* the largest reduction in pesticide suicide occurred, and paraquat, which accounted for the most pesticide-associated deaths, was not included [22, 23]. Some countries inadvertently experienced a change in paraquat-associated mortality as a result of fluctuations in the quantity of imported paraquat. When Western Samoa temporarily reduced paraquat importation due to national financial problems, paraquat use and self-poisoning ebbed with this change [14, 15]. When Suriname also limited paraquat importation in 1986 due to economic reasons, the monthly incidence of paraquat poisoning decreased in conjunction with its sparse availability to the general public [24].

The state of Andhra Pradesh in India was one case of a successful intervention. After the implementation of the non-pesticide management (NPM) policy, the suicide rate markedly decreased, which was in contrast to neighboring villages that continued using pesticides. However this study included too small sample size to conclude the effect of the intervention; 14 (before) to 3 (after) in number of pesticide-associated suicide) [25].

In Finland, the pesticide-related suicide rate due to the pesticide parathion rapidly increased during the 1950s, after the pesticide’s introduction. Due to subsequent restrictions of parathion’s availability in 1960s, the total suicide rate decreased. The decline occurred rapidly during first few years after the restrictions were imposed, suggesting that easy accessibility contributed to the use of parathion as a means of suicide. Later, when parathion became available only for those who stored parathion for its normal use, the pesticide-associated death rate decreased more slowly [26].

Our analysis of the National Death Certificates between 2009 and 2013 showed that the national ban on paraquat use in South Korea was an effective strategy. Most importantly, deaths from suicide significantly decreased in Korea after the national intervention. Cases of pesticide-associated deaths, which accounted for 9.7% of deaths from external causes in the before-intervention period, also decreased after the national ban on paraquat products. Deaths due to pesticide poisoning were considerably higher during the spring and summer months, seasons during which paraquat was widely spread to kill weeds. Previous studies have corroborated such seasonal variations [2, 27]. Individuals over 60 years old or with a low education level were more prone to pesticide poisoning. Blue collar occupations, including skilled agricultural and forestry workers, were highly associated with pesticide poisoning deaths. Our results were further substantiated by previous studies, which illustrated a remarkably high percentage of farmers possessing preexisting paraquat with respect to pesticide-associated mortality [28]. The rate of pesticide poisoning was significantly higher in suicide cases (15.1%), while pesticide-unrelated deaths predominated in accidental deaths (0.4%). According to Seok et al., only 30% of patients intentionally selected paraquat for self-harm and purchased paraquat products, while others used preexisting paraquat [28]. Thus, reduced exposure to paraquat seems to have contributed to the decrease in pesticide-associated deaths from external causes, as well as pesticide-associated suicides.

### Limitations

There were several limitations to this study. Because this was a before- and after-intervention observational study, unmeasured bias may have influenced the outcome. The outcome was defined as pesticide-associated mortality rather than herbicide or paraquat poisoning. Outcomes by pesticide were not associated with the intervention. However, we used pesticide-associated mortality rather than paraquat-induced mortality as a proxy because of the lack of precise information regarding specific poisoning agents on death certificates. Measuring the exact number of deaths by pesticide was technically impossible because in the national data pertaining to the ICD-10 classification, the category T603 includes both herbicides and fungicides. However, previous studies have indicated that paraquat was the most commonly used agent in pesticide-associated mortality in South Korea [2, 6, 7]. This study may have excluded more cases of survival after paraquat poisoning because they were not investigated, which may have contributed to selection bias. Furthermore, the effect of the intervention may have been overestimated due to the many public campaigns focusing on the ban of paraquat. For several months prior to the implementation of the policy, non-government organizations, academic societies, and media disseminated information about the ban on poison use in agriculture. These collaborative efforts and the subsequent policy implementation likely influenced the outcome. It is also difficult to estimate how much paraquat remained in circulation, as well as the frequency of illegal paraquat use, after enforcement of the policy. Because there are varying cultural, social, and economic factors influencing pesticide-associated deaths, additional individual and collective studies in other countries are needed to generalize our conclusions.

## Conclusion

The national public health intervention, which prohibited paraquat production and sales, significantly reduced pesticide-associated mortality in Korea. Among the intention groups, the effect was significant and pronounced in the Suicide and Undetermined groups.
